# Effects of glioblastoma-derived extracellular vesicles on the functions of immune cells

**DOI:** 10.3389/fcell.2023.1060000

**Published:** 2023-03-07

**Authors:** Oxana E. Musatova, Yury P. Rubtsov

**Affiliations:** ^1^ Shemyakin-Ovchinnikov Institute of Bioorganic Chemistry, RAS, Moscow, Russia; ^2^ N.N.Blokhin Russian Cancer Research Center, Ministry of Health of the Russian Federation, Moscow, Russia

**Keywords:** extracellular vesicles, immune response, signaling pathways, T cell response, glioblastoma multiforme, immune suppression

## Abstract

Glioblastoma is the most aggressive variant of glioma, the tumor of glial origin which accounts for 80% of brain tumors. Glioblastoma is characterized by astoundingly poor prognosis for patients; a combination of surgery, chemo- and radiotherapy used for clinical treatment of glioblastoma almost inevitably results in rapid relapse and development of more aggressive and therapy resistant tumor. Recently, it was demonstrated that extracellular vesicles produced by glioblastoma (GBM-EVs) during apoptotic cell death can bind to surrounding cells and change their phenotype to more aggressive. GBM-EVs participate also in establishment of immune suppressive microenvironment that protects glioblastoma from antigen-specific recognition and killing by T cells. In this review, we collected present data concerning characterization of GBM-EVs and study of their effects on different populations of the immune cells (T cells, macrophages, dendritic cells, myeloid-derived suppressor cells). We aimed at critical analysis of experimental evidence in order to conclude whether glioblastoma-derived extracellular vesicles are a major factor in immune evasion of this deadly tumor. We summarized data concerning potential use of GBM-EVs for non-invasive diagnostics of glioblastoma. Finally, the applicability of approaches aimed at blocking of GBM-EVs production or their fusion with target cells for treatment of glioblastoma was analyzed.

## Extracellular vesicles transfer information between cells

In multicellular organisms, cells mostly communicate with each other by exchanging chemical signals, or by direct contacts. Recently, more sophisticated way of communication was discovered. It uses small lipid bilayer-covered bubbles which may transfer cytosol from cell to cell. These vehicles, the so called extracellular vesicles (EVs), are considered as transporters of information and are extensively studied in the context of tumor microenvironment. EVs are produced by the most known cell types and are presented in essentially all body fluids from healthy donors and patients with various pathologies (Keller et al., 2011). Production of EVs is dramatically increased in tumor cells pointing to their potential involvement in interactions between tumor cells, tumor stroma and infiltrating immune cells. It was suggested that EVs can deliver membrane and/or cytosolic molecules and, hence, biological information from parent cell to its neighbors, or potentially to distant tissues and organs. EVs size ranges from dozens of nanometers to up to 2 μm depending on the origin (Muller et al., 2014). Small size allows them entering narrow gaps between cells, while lipid envelope protects their cargo from degradation in blood serum and other fluids (Benecke et al., 2021). Common classification and nomenclature of EVs have not been developed yet. The reasons are different mechanisms of EVs generation, complex composition and various isolation protocols. The prevailing class of EVs is exosomes that form upon fusion of multivesicular bodies (MVBs) or endosomes with plasma membrane (Bobrie et al., 2011). Microvesicles directly bud off from plasma membrane and are generally larger than exosomes. Another class of EVs are apoptotic vesicles and apoptotic bodies generated during apoptotic cell death. Exosomes are the most numerous, therefore, usually preparations of EVs are enriched with exosomes (Akers et al., 2013).

The subtypes of EVs also vary in size: exosomes are the smallest EVs (30–150 nm) and apoptotic bodies are the largest one (1000 nm or more). Microvesicles have intermediate size ranging from 100 to 350 nm. The division of EVs into exosomes, microvesicles and apoptotic bodies is based on the biogenesis pathway, nowadays this nomenclature is obsolete and not recommended for use (Sabbagh et al., 2020). The International Society for Extracellular Vesicles established a new classification of EVs depending on their size: small EVs (<200 nm) and large EVs (≥200 nm). This classification was established in 2018 and is still relevant (Bălașa et al., 2020). If it is impossible to determine the particle size, the term «extracellular particles» is preferable. The term «extracellular vesicles» certainly is also acceptable. Since the purity of biological samples cannot be fully ensure, the term «separation» is recommended instead of «isolation» or «purification» in case of vesicles (Théry et al., 2018). A more detailed description of vesicle biogenesis mechanisms is reviewed elsewhere (Teng and Fussenegger, 2020; Lombardi et al., 2021; Taylor et al., 2020).

EVs from different sources are unique in molecular composition which provides a great mean for defining their signature (van Niel et al., 2018). It makes EVs isolated from healthy donors and patients suitable for non-invasive diagnostics because amount of EVs and their composition may change alongside with disease development. By definition, EVs carry common surface markers participating in their formation, for example, tetraspanins, flotillin, Alix (ALG-2-interacting protein X) and other membrane proteins (Mathivanan et al., 2010; Thery et al., 2001, Tauro et al., 2012). Aside from these major constituents, more than 4400 proteins from EVs were extracted and identified (Mathivanan and Simpson., 2009). EVs contain sets of lipids such as cholesterol, ceramide and many others found in plasma membrane of parental cells (Subra et al., 2007). The outer surface of EVs is modified/contains sugars: mannose residues, α-2,6 sialic acid and branched N-linked glycans (Song et al., 2019). Essential components of EVs cargo are nucleic acids, in particular messenger RNAs (mRNAs) and non-coding RNAs (ncRNAs). Amount of these RNAs in EVs lumen can dramatically differ from that in cytosol of parent cells pointing to specific accumulation of some RNAs in EVs (Valadi et al., 2007).

According to literature, EVs affect target cells by binding to their surface, or they can fuse with the membrane of target cells, or endocytosed. Depending on the type of interaction, the effect of EVs on target cells can vary (McKelvey et al., 2015). For immune cells and tumor vesicles, it was demonstrated that EVs can both stimulate and inhibit lymphocytes depending on ratio and membrane composition. Effects of tumor EVs on immune cells were recently reviewed in (Ukrainskaya et al., 2019; Wang et al., 2022) Below we will discuss the role of EVs produced by glioma/glioblastoma in establishment of immune suppressive microenvironment.

## Glioblastoma: common facts

The most frequent type of the primary CNS tumors are gliomas—a group of heterogeneous malignancies that includes tumors of various origin, aggressiveness and growth rate. About half of them are glioblastoma tumors (GBM) that comprise about 80% of all brain tumors. GBM is highly aggressive with 5-year survival not exceeding 5% of patients (Ostrom et al., 2014a). The Central Brain Tumor Registry of the United States (CBTRUS) estimates the frequencies of gliomas and GBM as 6,61 and 3,19 per 100,000, respectively (Ostrom et al., 2014b).

According to Tamimi and colleagues, glioblastoma is often diagnosed in old patients with average age of 64 years. It is more frequent in males than females, at least in the United States, (3,97 *versus* 2,53 per 100,000, respectively) (Ostrom et al., 2014a). The highest occurrences of glioblastoma are reported in Western and Central Europe and North America, while the lowest - in Africa and some parts of Asia. This distribution suggests that genetic predisposition may affect development of glioblastoma (Omuro, 2013; McNeill, 2016; Patel et al., 2019).

Glioblastoma is characterized by increased mitotic activity, high invasiveness and development of necrotic zones. Increased phenotypic heterogeneity of the glioblastoma cells led to frequent addition of «multiforme» term in the medical literature. Glioblastoma contains cells of various differentiation states ranging from cells with low differentiation status along with highly differentiated subpopulations. In this regard, each patient with glioblastoma contains a unique set and proportions of heterogeneous tumor cells (Soeda et al., 2015).

Surgical resection is still the prevalent way of the GBM treatment, and complete removal of the glioma/GBM cells positively correlates with patients’ survival (Nieder et al., 2005). Often, glioblastoma treatment combines several complementary approaches: the surgical resection is usually followed by chemo- and/or radiotherapy. But even the combined protocols resulting in substantial reduction of the tumor volume do not affect generally poor outcome. Many glioblastoma patients survive for several years after successful treatment, while complete cure and long-term survival have to be achieved yet (Stupp et al., 2009). Unfortunately, early diagnostics and treatment do not affect the hopeless outcome questioning the usefulness of large-scale screenings for GBM. Better understanding of GBM development and progression should help developing specific and effective anti-glioblastoma therapy and improve grim prognosis.

## Protein components of GBM-EVs

**FIGURE 1:**
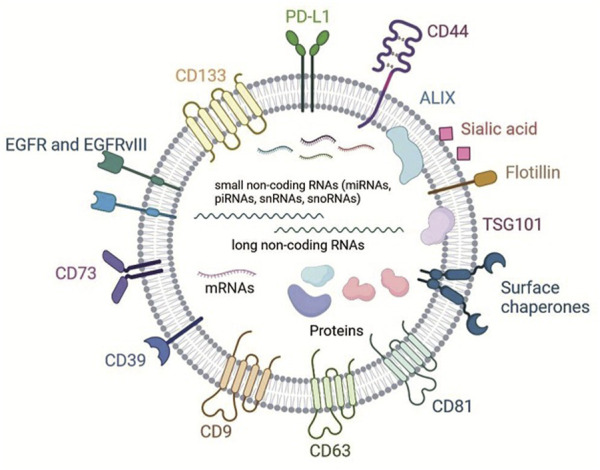
Scheme shows major protein membrane components and lipids of GBM-EVs and classes of cargo molecules (non-coding RNAs, mRNAs and cytosolic proteins, adopted with modifications from Benecke et al., 2021; Naryzhny et al. 2020).

EVs, as it was mentioned above, can be used by normal and tumor cells for intercellular communications. Therefore, molecular composition of the tumor EVs substantially differs from that of EVs obtained from normal non-tumor cells. GBM cells also produce EVs. Major set of surface proteins that was identified in GBM-EVs is presented in Figure 1. EVs from GBM (GBM-EVs) contain common vesicular markers: tetraspanins (CD9, CD63, CD81); flotillin-1, HSP70, Alix and Tsg101; and, in addition, GBM-EVs possess specific protein markers. For example, according to cytometric and western blotting analysis, GBM-EVs carry anti-inflammatory enzymes CD39 and CD73, both involved in tumor progression (Scholl et al., 2020). Proteomic studies found surface chaperones in isolated GBM-EVs, including HSP27 participating in cell differentiation and inhibition of apoptosis (Graner et al., 2009). Cell adhesion molecule CD44, known as one of the most common tumor markers, is overexpressed in GBM and is presented on the surface of GBM-EVs (Szatanek and Baj-Krzyworzeka, 2021). Non-differentiated cells from GBM secrete EVs with surface expression of prominin (CD133), commonly used as a marker of cancer stem cells (CSCs) (Thakur et al., 2021a). Receptor of epidermal growth factor (EGFR) is overexpressed in around 50%–60% cases of GBM, while its mutant variant, EGFRvIII, is present in ∼50% of GBM. Both proteins are poor prognostic factors, especially in patients younger than 45 (Heimberger et al., 2005). EGFRvIII can be easily found in cell lysates and is present in GBM-EVs (Al-Nedawi et al., 2008; Graner et al., 2009; Ricklefs et al., 2016). Anti-inflammatory molecules PD-L1 (programmed death ligand -1) and IDO1 (indoleamine-2,3-dioxygenase 1, the enzyme which degrades extracellular tryptophan) are also both markers of GBM cells and EVs, these proteins are upregulated following the treatment of GBM cells with IFN-γ (gamma interferon, key anti-tumor proinflammatory cytokine). PD-L1 and IDO1 play significant role in tumor progression which will be discussed further (Jung et al., 2022). Most of these EV surface molecules are specific for tumor cells including GBM, and could be used as a GBM markers as will be discussed below.

Composition of protein cargo of GBM-EVs also has specific features. 133 proteins were identified in proteomes of EVs from five primary GBM cells lines according to Naryzhny et al. 2020 (Figure 1). They were annotated and clustered according to the function. Large group of proteins include structural vesicular proteins, for example, clathrin (CLTC); chaperones, components of cytoskeleton, small G-proteins and vimentin (VIME). Separate group consists of polypeptides that interact with nucleic acids—components of chromatin and ribosomal proteins. Members of another important cluster include GBM-EVs proteins participating in metabolism—enzymes, ionic channels, transporters of amino acids and subunits of G-proteins regulating adenylate cyclase and phospholipase C. Authors highlighted a set of proteins supporting GBM survival. They include lactate dehydrogenase B (LDHB) and phosphoglycerate kinase 1 (PGK1) which promote adaptation of cells to shortage of energy; catalytic subunit of DNA-dependent protein kinase (PRKDC), major vault protein, and proteins involved in DNA repair (Naryzhny et al., 2020). Other study reported a transfer of transglutaminase responsible for increase of mitogenic signaling in cells treated with GBM-EVs (Antonyak et al., 2011), and TGF-β (transforming growth factor beta), the cytokine with potent immune suppressive activity (Graner et al., 2009) in primary GBM cell lines. These proteins are not GBM-specific, but they are frequently overexpressed in GBM. The presence of proteins participating in metabolism, DNA reparation and mitosis in EVs factors in establishment of tumor microenvironment (TME).

## RNAs from GBM-EVs

### mRNA

RNA molecules from GBM-EVs draw major interest because they supposedly should possess regulatory potential and induce functional reprogramming or differentiation of target cells. The analysis of both EVs from patient-derived GBM cells and patient serum (Skog et al, 2008) identified about 27,000 species of mRNA. Interestingly, around 4700 of them were found only in GBM-EVs, and 3000 of these mRNAs differed from that in parental cells (2238 mRNAs were enriched in EVs, and 1188 were almost excluded from EVs). The majority of GBM-EVs mRNAs were associated with the following functions: angiogenesis, cell proliferation, immune response, migration of cells and histone modifications. It should be noted that RNA isolated from EVs circulating in the blood of patients with GBM multiforme had reduced levels of RNAs involved in formation of ribosomal subunits compared to healthy donors (Noerholm et al., 2012). We summarized the information concerning major RNA and DNA components of GBM-EVs in Supplementary Table S1. Collectively, these data indicate non-random loading of mRNA into GBM-EVs suggesting enrichment of RNA with potential regulatory functions.

### microRNA

**FIGURE 2:**
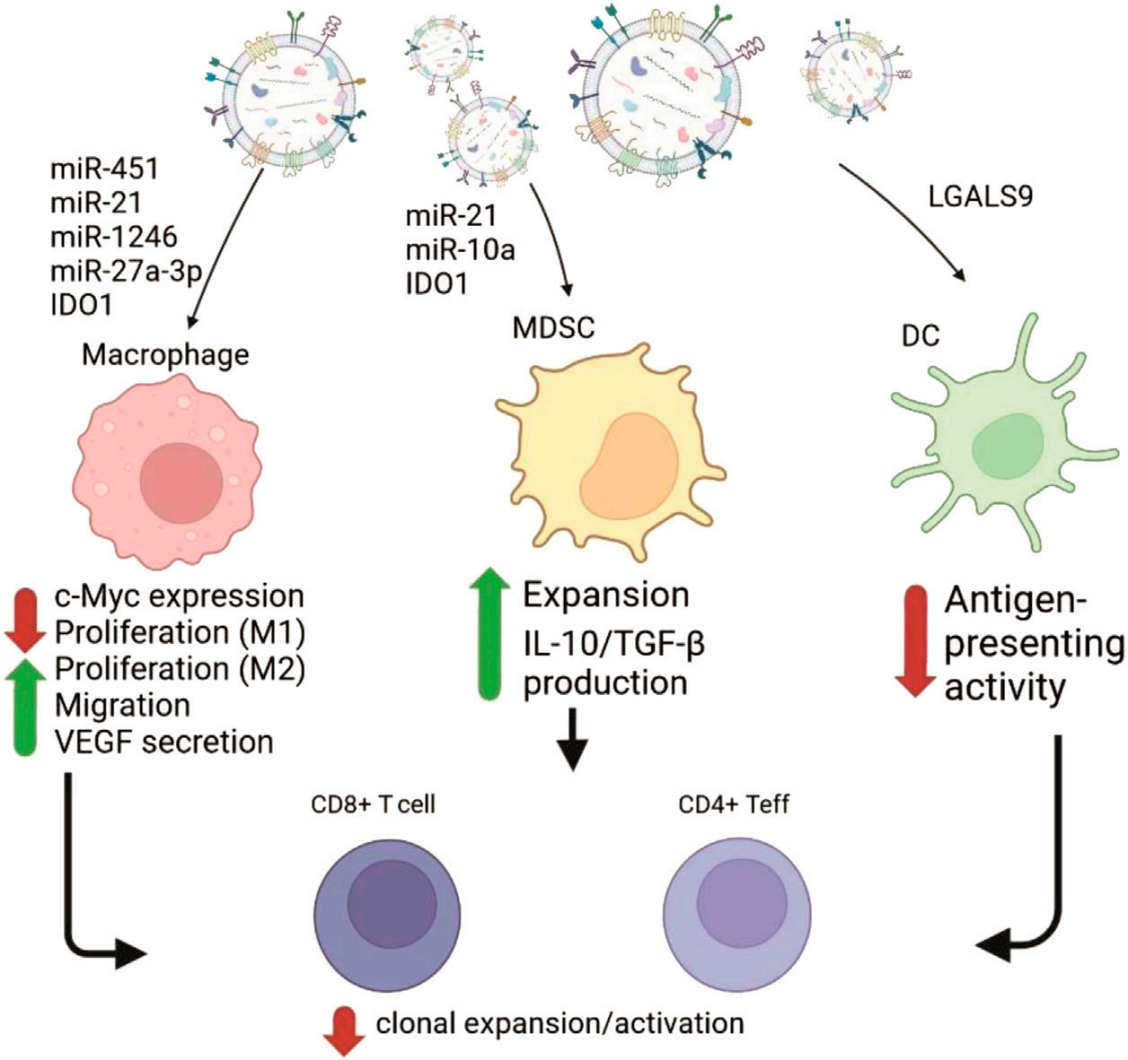
Schematic representation of GBM-EVs effects on the myeloid immune cells mediated by transfer of the cargo molecules (mostly RNAs). GBM-EVs (on the top) fuse with or get phagocytosed by macrophages, myeloid-derived suppressor cells (MDSC) and dendritic cells (DC) causing changes in phenotype of recipient cells. Cargo molecules participating in reprogramming and resulting phenotypic responses (Cheryl et al., 2013; Mooij et al., 2020; Liang et al., 2019; Jung et al., 2022) are shown as red (negative effect) or green (positive effect) arrows with supporting text. Subsequent influence of changes in myeloid cells negatively affect anti-tumor immunity by suppressing functions of CD4^+^ effector cells and CD8^+^ cytotoxic lymphocytes (on the bottom).

Analysis of microRNAs (miR) from GBM-EVs obtained from human U251 GBM cell line also demonstrates dramatic discrepancy of their levels with that in the source cells. This suggests specific production and packaging for export of multiple miR species in tumor cells. GBM-EVs are enriched with several oncogenic miRs including miR-21, miR-10a, miR-23a, miR-30a, miR-221 and miR-451 (Supplementary Table S1; Cheryl et al., 2013). Transfer of these regulatory molecules between tumor and normal cells could be possibly critically involved in growth, invasion and survival of tumor cells. Van der Vos and colleagues confirmed that miR-451/miR-21 can be transferred from GBM cells to microglia *in vivo* (Van Der Vos et al., 2015). The transfer of immune suppressive miR-21 by EVs from five low passage human GBM cell lines was also shown in other study along with delivery of other miRs: let-7, miR-3182, miR-4448, miR-100-5p, miR-27-3p (Mooij et al., 2020). Information concerning the influence of miR loaded into EVs from glioblastoma on myeloid cells and the consequent modulation of T cell functions is shown in Figure 2.

Under hypoxic stress conditions, several miRs were upregulated in GBM-EVs. The list includes miR-210, miR-1275, miR-376c, miR-23b, miR-193a and miR-145. It is known that increased production of miR-210 during hypoxia results in elevated synthesis of vascular endothelial growth factor (VEGF) by human GBM U87 and U251 lines supporting survival of cells (Agrawal et al., 2014). This fact points to the possibility of positive influence of vesicular transfer of miR-210 on survival of GBM cells under the hypoxic conditions (Figure 2). Above, the set of miRs which level is higher in GBM-EVs than in GBM cells were discussed. But there is a set of miRs underrepresented in the GBM-EVs in comparison to GBM cells. Mostly, their function relates to inhibition of cell growth and tumor progression. For example, ectopic expression of tumor suppressor miR-1 in GBM results in reduction of growth rate, invasion and neovascularization. This effect was partially explained by increased incorporation of overexpressed miR-1 into GBM-EVs. Aside from effect of miR-1 from GBM-EVs on GBM cells, this inhibitory RNA targets prooncogenic signaling pathways in cells forming GBM microenvironment. Transfer of functional miR-1 using GBM-EVs to other target cells leads to downregulation of its target mRNAs *in vivo* (Bronisz et al., 2014; Rooj et al., 2016).

### Long non-coding RNA

Aside from miRs, non-coding RNAs are presented in GBM cells and GBM-EVs by a diverse class of long non-coding RNAs (lncRNAs). We are not going to discuss biogenesis and functions of lncRNAs because there are many excellent reviews on this topic (Yadav et al., 2021; Nandwani et al., 2021; Taniue and Akimitsu., 2021). lncRNA HOTAIR is involved in development of different tumors. In gliomas it acts as oncogene and displays angiogenic function (Ma et al, 2017). lncRNA ROR1-AS1 facilitates glioma progressing when overexpressed and transferred from cell to cell by EVs. ROR1-AS1 has a function of sponge RNA for miR-4686 which inhibits tumor progression (Chai et al., 2020). ROR1-AS forms complementary duplexes with miR-4868 and decreases amount of free miR causing de-repression of its target genes and promotes tumor growth. Expression of lncRNA SBF2-AS1 correlates with resistance of GBM cells to temozolomide (TMZ) and poor prognosis, while its overexpression makes cells more resistant to this chemotherapeutic drug. EVs produced by cells overexpressing SBF2-AS1 have high level of this lncRNA and converted TMZ-sensitive GBM cells to TMZ-resistant. (Zhang et al., 2019).

#### Other classes of ncRNA

Analysis of RNAs isolated from EVs circulating in peripheral blood of GBM patients suggests that they carry excessive amounts of uncharacterized RNA shorter than 500 nucleotides (Noerholm et al., 2012). Many of them are new, and their function have not been identified. Almost half of these RNAs belongs to a class of new small RNAs which can be mapped in intronic and intergenic regions and encoded by both sense and antisense strand of genomic DNA. Majority of these RNAs were not detected in parenting cells pointing to their role as export cargoes (Cheryl et al., 2013). Most RNAs from this pool were annotated as piRNAs, snRNAs, snoRNAs and yRNAs (Mooij et al., 2020). The variety of circRNAs were also reported to be as a part of specific RNA cargo of GBM EVs (Wang et al., 2021a; Ding et al., 2019). These classes of RNAs are involved in regulation of gene activity either directly or indirectly by affecting functions of miRs.

#### Mobile elements and genomic repeats

Special attention was attracted to another class of nucleic acids contained in GBM-EVs and represented by repeating DNA elements of the human genome. It was found that sequences from SINE repeats, LTRs and human endogenous retroviruses (HERV), Alu and L1 repeats can be identified in isolates of nucleic acids from GBM-EVs secreted by primary GBM cell lines (Supplementary Table S1). Authors suggest that repeats could participate in silencing of genes and genomic translocations in cells receptive to EVs. Specificity and magnitude of effects caused by multiple repeating elements is questionable, but authors speculate that transfer of genomic repeats causes transformation of cells and development of tumors (Cheryl et al., 2013; Balaj et al., 2011).

## Effects of GBM-EVs on TME: The non-immune participants

There are indications that distinctive molecular pattern characteristic for GBM-EVs is required to support tumor development and growth. Exchange of vesicles containing oncogenic and transforming factors help spreading tumor to new niches (Chen et al., 2021). These niche-colonizing signals could be delivered either by direct membrane contacts between EVs and recipient target cells, or by internalization of EVs inner content (Katakowski et al., 2010; Ridder et al., 2015). Exchange of vesicles can happen between different tumor cells, or alternatively, between tumor cells and normal cells of surrounding tissue. These events can lead to functional reprogramming (Vlassov et al., 2012). For example, EVs from human GBM line U87 can impart properties of transformed and tumor cells such as uncontrolled proliferation and increased survival to normal fibroblasts and epithelial cells (Antonyak et al., 2011). Normal astrocytes demonstrated increased migration rates and elevated secretion of cytokines and growth factors following treatment with tumor EVs (Oushy et al., 2018). In this context, cytokines can synergize the effects of EGF on recruiting of precursor cells of mesenchymal origin (Birnbaum et al., 2007; Schichor et al., 2006). Highly beneficial effects on GBM development were attributed to adipocytes. They can act distantly by producing EVs increasing the size of glioma tumors and inhibiting apoptosis of glioma cells *in vivo* (Del Fattore et al., 2014). Exchange of tumor vesicles between cancer cells also promotes tumors. Incorporation of EGFRvIII from EVs membranes to plasma membrane of U373 cell line causes increased production of VEGF and boosts expression of anti-apoptotic protein Bcl-xL (Al-Nedawi et al., 2008). Apoptotic GBM-EVs which were secreted after experimental treatment of primary GBM cell lines with chemo- or radiotherapy are ingested by surviving GBM cells which acquire more aggressive, highly invasive and therapy-resistant phenotype. This observation possibly explains very high level of post-treatment relapses in the case of GBM (Pavlyukov et al., 2018).

## Interaction of GBM with immune system

**FIGURE 3:**
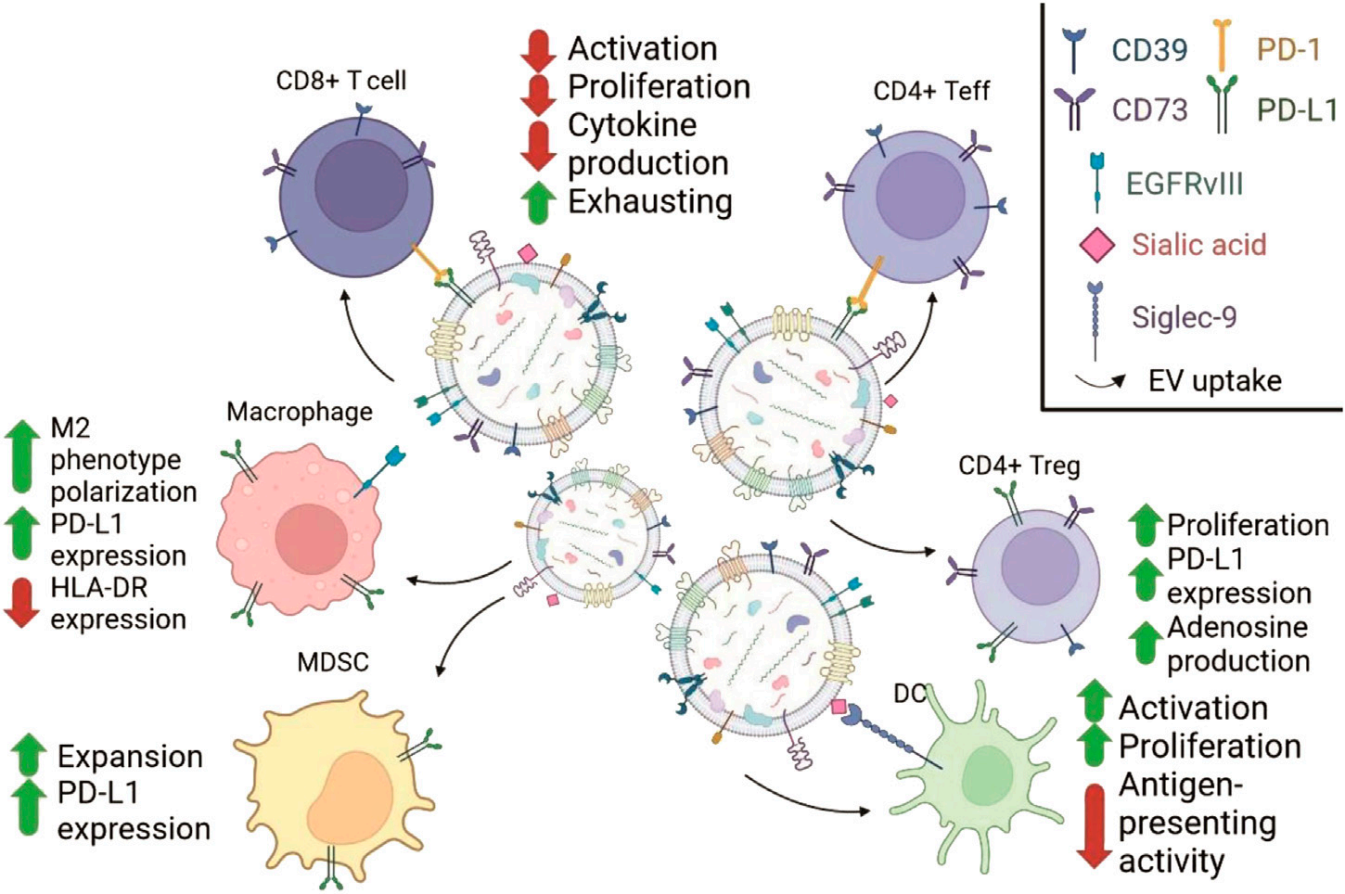
Schematic representation of GBM-EVs effects on the immune cells mediated by the surface molecules. GBM-EVs (in the middle) carry molecules participating in ligand-receptor interactions with molecules on the surface of immune cells (adopted with modifications from Scholl et al., 2020; Ricklefs et al., 2016; Jung et al., 2022; Dusoswa et al., 2019). The changes in immune cells are indicated with arrows with supporting text. Chart in the upper right corner explains the symbols used to show different membrane proteins from GBM-EVs.

Despite immune privileged localization, GBM eventually meets immune cells following breaching of the blood brain barrier. Usually, about 1% of GBM cells are CNS macrophages and microglia which possess peculiar phenotypic features. They express high levels of TLRs but fail to proliferate and secrete proinflammatory cytokines following stimulation with TLR ligands. Macrophages and glia from GBM express MHCII on the surface but lack co-stimulatory molecules CD86, CD80 and CD40 required for effective activation of T cells (Hussain et al., 2006). GBM-infiltrating macrophages also express approximately 5 times less miR-142-3p in comparison to normal brain macrophages (Xu et al., 2014). Since in GBM anti-inflammatory CD11b^+^CD163^+^ macrophages have decreased level of miR-142-3p in comparison to antitumor pro-inflammatory CD11b^+^CD163^−^macrophages, this points to overall anti-inflammatory environment in GBM (Attaran and Bissell., 2022). Summary of effects that could be attributed to GBM-EVs effects on various cells of immune system is shown in Figure 3.

Another tumor-infiltrating cell type that should recognize tumor antigens and induce tumor killing are T cells. Sometimes, tumor infiltrating lymphocytes’ (TILs) numbers can reach 300 cells per about 100 tumor cells, and their pool contains clones not detectable in circulating blood (Perrin et al., 1999). Large numbers of CD8^+^ T cells which supposedly should kill tumor was not sufficient to inhibit tumor due to the deficit in functional CD4 helper cells (Miggelbrink et al., 2021). These dysfunctional CD8^+^ cells are actively dividing and prone to spontaneous apoptosis (Yu et al., 2003), or fail to undergo activation (Hussain et al., 2006). Summary of effects that could be attributed to GBM-EVs effects on various cells of immune system is shown in Figure 3.

CD4^+^ TIL from GBM contain a large fraction, up to 50%, of CD56^+^ T cells. Proportion of proliferating CD56^+^CD4^+^ T cells was 3–4 times higher than fraction of proliferating CD56^−^cells, and major fraction of CD56^+^ cells produced Th2 cytokines IL-4 and IL-13 (Waziri et al., 2008; Belghali et al., 2022). Another study reports massive infiltration of CD4^+^FoxP3^+^CD25^high^CD127^low^ regulatory T cells (Treg) in GBM and other metastatic brain tumors (Hussain et al., 2006). Brain tumor Tregs are characterized by increased level of CTLA-4 and FoxP3 (forkhead-box winged helix P3) in comparison to blood Treg. In most cases tumor Treg in GBM were localized in a close proximity to effector cells (Jacobs et al., 2009).

From the facts presented above, it is logical to assume that GBM creates potent immune suppressive TME. GBM uses several ways to establish and cement immune suppressive conditions: it can recruit cells secreting anti-inflammatory cytokines; (Waziri et al., 2008; Tamai et al., 2022), or it overexpresses ligands of immune inhibitory receptors such as PD-L1, HLA-E/HLA-G on the surface (Mittelbronn et al., 2007; Wiendl et al., 2002; Jacobs et al., 2009). In addition to this arsenal, GBM cells often express FasL directly inducing apoptosis of activated tumor-specific T cells (Husain et al., 1998; Frankel et al., 2000). Apoptotic Fas-positive T cells can be often found close or in contact with tumor cells expressing FasL (Didenko et al., 2002; Saas et al., 1997).

## GBM-EVs inhibit anti-tumor immunity

GBM-EVs play critical role in suppression of anti-tumor immune response. They do it by a various means, and immune suppressive mechanisms make complex TME even more complicated. The main result of interactions between GBM-EVs and TILs is functional exhaustion and inability of T cells to kill the tumor (Dunn et al., 2007). Below we will discuss the most potent mechanisms of immune suppression mediated by GBM-EVs and directed towards T cells or immune suppressive subtypes of tumor-infiltrating cells (Figure 2, Figure 3).

### PD-L1/PD-1

GBM usually secrete EVs with high level of PD-L1. This Ig-like molecule binds its ligand PD-1 located on the surface of activated T-cells. Stimulation of PD-1 recruits tyrosine phosphatases SHP-1/-2 to phosphorylated ITAM motifs. As a result, TCR’s components become de-phosphorylated and do not transfer activating signals to downstream molecules. Thus, PD-L1 on the GBM-EVs blocks activation and proliferation of T cells caused by stimulation of TCR (Salmaninejad et al., 2019). It was shown, that PD-L1 from EVs directly binds PD-1 on T cells. PD-L1 level in GBM correlates with aggressiveness and surface phenotype. PD-L1-rich GBM variants usually have mesenchymal phenotype, while PD-L1^low^ cells demonstrate less aggressive pro-neural phenotype. Since tumor could contain both mesenchymal and pro-neural cells, T cell inhibition was expected to be more profound by GBM with predominantly mesenchymal phenotype due to high level of PD-L1 on the EVs (Doucette et al., 2013). However, EVs from GBM stem cells (GSCs) with high and low PD-L1 level similarly strongly inhibit T cell activation according to changes in early and late activation markers CD69 and CD25. It was suggested that EVs with low PD-L1 utilize the molecular mechanism distinct from PD-L1/PD-1 signaling. Uptake of these PD-L1^low^ EVs was found to increase the levels of IDO1 and IL-10 mRNAs in recipient cells; both mRNAs encode immune suppressive proteins. Interestingly, the treatment of GBM cells with low PD-L1 with IFN-γ resulted in upregulation of PD-L1 on the cell surface and on the EVs, indirectly confirming importance of PD-L1 in GBM-EVs-mediated immune suppression (Figure 3; Ricklefs et al., 2018).

### Extracellular enzymes

GBM-EVs carry two nucleosidases - CD39 and CD73—on the outer side of membrane (Scholl et al., 2020). These enzymes convert extracellular ATP to adenosine. CD39, first, hydrolyzes ATP to ADP and 5′-AMP, which is then further degraded to adenosine by CD73. The shift from ATP to adenosine changes extracellular pro-inflammatory ATP rich milieu to adenosine high anti-inflammatory environment. High levels of CD39 and CD73 can be detected on activated T lymphocytes, especially, on CD4^+^ and CD8^+^ T cells, and in CD4^+^Foxp3^+^ Treg that normally suppress excessive immune responses. Subpopulation of Treg with distinct transcriptomic signature is attracted by various tumors to inhibit specific anti-tumor immune response (Bavaresco et al., 2008). Elevated expression of CD73 was described in various malignancies, for instance GBM, and usually is associated with poor prognosis. CD73 can be transported by EVs to T cells. Uptake of vesicular CD73 by lymphocytes limits their expansion initiated by stimulation with anti-CD3/CD28 mAbs. CD73 from EVs changes energy consumption by T cells. This enzyme downregulates glucose transporters Glut1 and Glut3 and inhibits expression of several enzymes in aerobic glycolysis cascade (Ohta et al., 2006). But main suppressive effect of CD73 is caused by conversion of 5′-AMP to adenosine. Adenosine is a signaling mediator which binds to isoform of adenosine receptor A2AR. Signaling through A2AR increases production of cyclic AMP and dephosphorylation of phosphoSTAT5 (Signal transducer and activator of transcription 5). These events negate signals from IL-2 receptor and TCR in T cells. Involvement of adenosine in immune suppression was indirectly confirmed by elevated levels of adenosine in GBM patients and mice with transplanted GBM tumors (Wang et al., 2021b).

### Other molecules

Proapoptotic surface molecule FasL limits T cell responses by induction of apoptosis during direct contacts between cell with surface Fas (usually activated T cells) and cells/EVs which expose FasL. Several types of brain tumors, for example, astrocytoma and oligodendroma produce EVs that carry FasL on their surface (Frankel et al., 2000; D'Agostino et al., 2006). GBM also expresses FasL on the surface (Husain et al., 1998). It was reported that GBM-EVs secreted by glioma model cell line GL26 promote the growth of implanted tumor and inhibited cytotoxic activity of CD8^+^ T cells both *in vitro* and *in vivo.* Also, they negatively affected the proportion and number of splenic CD8^+^ cells, this effect was accompanied by decreased secretion of IFN-γ and granzyme B. Authors of this study suggest involvement of Fas/FasL in induction of apoptosis in T cells *in vitro* and *in vivo* (Liu et al., 2013). In other work, preincubation of naïve T cells (as fraction of PBMC) with EVs from medulloblastoma cell line resulted in diminished activation by phytohemagglutinin (PHA) detected by downregulation of CD69 and IFN-γ production. These phenotypic changes were induced by low doses of EVs (100 mg/mL) but were more dramatic at higher doses (2000 mg/mL). Even very low doses of EVs (5–50 mg/mL) were capable to induce exhausted phenotype in T cells during activation (Epple et al., 2012). Despite expectations, the authors failed to detect FasL in cells and EVs preparations using western blotting (Hellwinkel et al., 2015). These data hint that FasL could be transferred by GBM-EVs to target cells, but direct evidence is missing.

Oligodendroma can make EVs that transport other pro-apoptotic molecule—the tumor necrosis factor (TNF)-related apoptosis-inducing ligand (TRAIL). 25%–50% astrocytes, treated with these EVs had signs of cell damage (Di Liegro et al., 2011). In GBM, this mechanism has not been reported yet.

There are implications that vesicular LGALS9 (Galectin-9), the ligand of CD4 T cell surface molecule TIM-3 (T cell immunoglobulin and mucin domain containing-3), is also involved in EVs-mediated immune suppression. Binding of Gal-9 to TIM-3 on T cells results in T cell apoptosis. High level of LGALS9 is an indicator of bad prognosis for all stages of glioma (Liang et al., 2019) It should be noted that LGALS9/TIM3 signaling pathway regulates T cell functions in several different ways such as regulation of apoptosis in CD4^+^ T cells and functional exhaustion of CD8^+^ T cells (Wang et al., 2020).

Once absorbed, GBM-EVs could also influence the immune response by passing cargo molecules to recipient cells. GBM-EVs contain TGF-β which was reported to be transferred in vesicles and directly inhibits the function of T cells (Graner et al., 2009). Identification of GBM-EVs cargo proteins which interact with nucleic acids could indicate that fusion with EVs possibly interferes with transcription of genes in recipient immune cells. Other cargo proteins such as enzymes, ion channels, transporters of amino acids, and G-proteins participating in metabolic processes could likely change the metabolic pathways (Naryzhny et al., 2020). GBM-EVs were also reported to contain spliceosomal proteins and snRNAs which affect mRNA splicing in recipient cells (Pavlyukov et al., 2018).

The transcripts of immune inhibition proteins in vesicular mRNAs pool (for example, leukocyte elastase inhibitor and *homo sapiens* cytokine receptor-like factor 1) were found to be selectively loaded into the EVs from GBM patients (Noerholm et al., 2012). miR-10a and miR-21 are involved in RORα and Pten signaling and activate myeloid-derived suppressor cells which affect function of T cells (Guo et al., 2018). Repeating and mobile genome elements were reported to cause multiple mutations in recipient cells disrupting vital functions (Cheryl et al., 2013; Balaj et al., 2011). All these cargo molecules inhibit T cell anti-tumor immune response through disturbance of normal biological processes inside the recipient cells and changing signaling pathways (Naryzhny et al., 2020).

## GBM-EVs target cell types involved in suppression of immune response

As it was mentioned before, TME is formed by multiple cell types including tumor cells itself, vascular cells, stromal cells and immune cells. Some of them are directly involved in immune suppression and are recruited by tumor to shield off immune response. Recruiting of suppressors and instructing their precursors by TME, for example, by interaction with GBM-EVs is likely a key event in tumor immune evasion (Hussain et al., 2006; Attaran and Bissell., 2022). The most studied cell types affected by GBM-EVs and having potent immune suppressive activity include the abovementioned tumor Treg, tumor macrophages, myeloid-derived suppressor cells (MDSC) and tumor dendritic cells (DCs). The most important data concerning effects of GBM-EVs on these adverse cells will be discussed below.

### Tumor Treg

Recruiting of Treg by tumors is a critical step in establishment of TME. They accumulate in large numbers in the tumors of different origin and support/provide potent immune suppression. Tumor Treg express chemokine receptor 4 (CCR4), while its ligand CCL12 (chemokine ligand 12) is secreted by GBM that could explain infiltration of tumor by Treg (Jacobs et al., 2010). Earlier studies demonstrate that TIL contain higher proportion of Treg than T cells from CNS of healthy individuals (Heimberger et al., 2008). At the same time, proportion of CD4^+^ T cells among TILs was gradually declining with time probably suggesting decreasing of CD4^+^ effector T cells. Proportion of Treg in the spleen of mice with transplanted gliomas was markedly lower in animals with tumors in comparison to healthy mice, these splenic Treg were enriched with cells expressing immune suppressive cytokine IL-10 (Figure 3; Kennedy et al., 2009).

Massive infiltration of Treg with surface phenotype CD4^+^FoxP3^+^CD25^high^CD127^low^ in GBM and metastatic brain tumors was found in other study. Authors report that tumor-infiltrating Treg express higher levels of CTLA-4 and Foxp3 than blood Treg from the same patients. The majority of tumor Treg, according to immunohistology of tumor sections, were localized in proximity from effector T cells (Jacobs et al., 2009).

GBM-EVs could mediate the recruiting of Treg and other suppressor cells to GBM tumors (Epple et al., 2012), but the most effective recruiting molecule for Treg is CCL12. Consumption of CD39^−^and CD73^−^expressing GBM-EVs by murine Treg results in increased production of adenosine and defects in energy generation pathways in effector T cells (Wang et al., 2021a). PD-L1 from GBM-EVs obtained from primary GBM lines participates in conversion of naïve and T helper cells to Treg, while expression of PD-1 and PD-L1 in Treg regulates their ability to inhibit function of effector T cells. At the same time, incubation of purified activated T cells with GBM-EVs did not result in induction of Treg phenotype (Li et al., 2017). Moreover, multiple reports (Himes et al., 2020; De Vrij et al., 2015; Jung et al., 2022) failed to detect direct inhibition of T cells or induction of apoptosis by GBM-EVs. In several reports, it was shown that tumor EVs and GBM-EVs had positive effect on proliferation of purified CD4^+^ T cells (Domenis et al., 2017; Ukrainskaya et al., 2021). It is likely that other populations of immune cells act as mediators between GBM-EVs and T cells, or depending on the amount of the EVs, they may modulate functions of both effector and suppressor T cell subpopulations.

### Macrophages and microglia

The majority of cells from proinflammatory infiltrate of the CNS tumors are microglia and macrophages. In healthy humans’ CNS, these cells are the main innate immune cells maintaining immune homeostasis. Microglia and several populations of CNS macrophages derive from precursors starting from embryonic stages of development. In case of infection, microglia and macrophages switch to proinflammatory state and secrete cytokines to induce cytotoxic response against invading microbes (Tamai et al., 2022). The situation is complicated by the fact that monocytes can migrate to CNS where they differentiate to macrophages in adults with neurotrauma (Kumar et al., 2015). According to simplified classification, macrophages belong to either M1 or M2 polarized cells which produce different sets of cytokines and support proinflammatory or anti-inflammatory conditions. M1/M2 classification is simplified and not recommended for use. It is better to use the combination of genes and/or protein markers to describe the chosen macrophage population (Paolicelli et al., 2022). Nevertheless, the M1/M2 classification is still widely used as it takes time for new one to come into use.

The macrophages uptake pathogens by phagocytosis and participate in tumor surveillance (Poon et al., 2017), therefore inhibition of their function is the other way of immune evasion by GBM. Macrophages and microglia sponge GBM-EVs when they are added to unfractionated PBMC. This phenomenon accounts for decreased influence of GBM-EVs on T cells in such experiments. Several studies showed fast adsorption of EVs by monocytes (Di Trapani et al., 2016) as well as efficient uptake of GBM-EVs from primary cell lines by microglia leading to increased proliferation and skewing of cytokine profile to immune suppressive (Van Der Vos et al., 2015). Since GBM-EVs cargo is enriched with proteins interacting with extracellular matrix and affecting cell migration, their uptake by monocytes changes the differentiation of the latter. Incubation of PBMC with EVs from U87 human GBM cell line results in dramatic increase in expression of markers CD14, CD16, CD32, CD45, CD163; and increased secretion of IL-6, MCP-1 and VEGF (Azambuja et al., 2020; Xu et al., 2021a). Likewise, EVs isolated from the cultures of primary GSC supported the differentiation of blood monocytes to anti-inflammatory macrophages. EVs from GSC increased expression of membrane type 1-matrix metalloproteinase (MT1-MMP, tumor-promoting factor and marker of glioblastoma-associated microglia) upon incubation with primary human microglia. Overall, EVs from GBM of mesenchymal subtype provided stronger effect on differentiation of monocytes to CD11b^+^CD163^+^ macrophages and increase in secretion of VEGF, growth factor having a role in progression of mesenchymal GBM (De Vrij et al., 2015). Deregulation of macrophage polarization by GBM-EVs is further supported by accumulation of CD11b^+^CD14^+^CD163^+^ macrophages. CD163 is a scavenger receptor which senses both gram-negative and gram-positive bacteria (Fabriek et al., 2009). High levels of CD14^+^CD163^+^ monocytes in circulation is often detected in GBM patients; in combination with elevated levels of serum IL-4 and IL-13. Th2 cytokines produced by CD163^+^ monocytes are reliable indicators of the immune deregulation by GBM (Harshyne et al., 2015). Taken together, skewing of macrophage polarization towards pro-oncogenic phenotype is the main strategy used by GBM-EVs to combat immune system.

Van der Vos and colleagues described another mechanism of macrophage suppression in GBM. They confirmed GBM-EVs-mediated transfer of miR-451 and miR-21 to microglia leading to downregulation of their target RNA, c-Myc. GBM-EVs were isolated from primary GBM cell lines (Van Der Vos et al., 2015). c-Myc controls multiple genes involved in proliferation. In cancers this particular mRNA is usually overexpressed but decrease in c-Myc mRNA in macrophages and microglia helps inhibiting anti-tumor immunity (Esser et al., 2020). EVs from established glioblastoma cell lines can also directly negatively affect expression of HLA-DR (human leucocyte antigen DR isotype) in macrophages (Iorgulescu et al., 2016) and, therefore, limit their antigen-presenting capacity. In addition, treatment of macrophages with GBM-EVs from patients’ blood and primary GBM cell lines resulted in almost complete loss of co-stimulatory molecules CD86, CD80 and CD40 (Hussain et al., 2006). These changes in macrophages following EVs treatment critically decrease their ability to activate T cells.

Local and systemic suppression of T cell responses by anti-inflammatory macrophages largely depends on secretion of immune suppressive cytokines such as IL-10. But under the influence of the EVs suppressive modes can change. For example, PD-L1 delivered by EVs could be passed to macrophages and other cells. In TME PD-L1 can be found on macrophages and microglia, these cells by themselves could be a source of vesicle-associated PD-L1 (Ricklefs et al., 2018). This PD-L1 of EVs origin also can positively affect differentiation of monocytes to anti-inflammatory CD11b^+^CD163^+^ macrophages (Himes et al., 2020; Jung et al., 2022). It was shown that monocytes pre-cultured with GBM-EVs significantly inhibit proliferation of T cells. PD-L1 and IDO1 are required for this monocyte-mediated suppression. Interestingly, the decrease in T cell proliferation was even more substantial when monocytes were cultured with EVs isolated from cultures of IFN-γ-treated GMB cells. This finding is in accordance with the fact that IFN-γ boosts production of PD-L1 by GBM cells and increases PD-L1 level in the GBM-EVs. Two-fold increment in CD14^+^/PD-1^+^/CD16^+^/HLA-DR^high^ macrophages in monocyte cultures treated with PD-L1-enriched GBM-EVs confirms that PD-L1 participates in alternative polarization of macrophages. EVs were isolated from low passage stem-like GBM lines (Jung et al., 2022).

### Myeloid-derived suppressor cells (MDSC)

MDSC are suppressor cells of myeloid origin, they are highly heterogeneous, actively proliferate in various pathologies including malignancies and, according to surface phenotype, resemble granulocytes (Himes et al., 2020). MDSC promote tumor angiogenesis, increase drug resistance, metastasis and systemic immune suppression. In some aspects, MDSCs’ phenotype is similar to that of tumor-infiltrating M2 macrophages, namely, they are CD14^high^/HLA-DR^low^. MDSC can be usually found in monocytic fraction of circulating cells in GBM patients and is associated with poor prognosis (Jung et al., 2022).

Along with other types of immunocytes, MDSC are also affected by GBM and GBM-EVs. Treatment of PBMC with GBM-EVs *in vitro* increases amount of MDSC approximately 1,5-fold. GBM-EVs from cultures treated with IFN-γ increase MDSC level even more potently—up to three-fold (Jung et al., 2022). Accordingly, MDSC from these cultures have more profound immune suppressive phenotype and changes in the miR profile (Ridder et al., 2015).

MDSC suppress immune responses by secreting anti-inflammatory IL-10 and TGF-β cytokines, but uptake of GBM-EVs may change the mechanism of immune suppression by MDSC. Similar to macrophages, induction of MDSC requires PD-L1 and IDO1, delivered by GBM-EVs (Jung et al., 2022). The rate of EVs uptake by MDSC is comparable to that of microglia and macrophages; MDSC can also serve as a source of PD-L1-loaded EVs (Ricklefs et al., 2018). It was shown that under hypoxic conditions GBM secretes larger numbers of EVs which differ from normal GBM-EVs according to their mRNA profile. These so-called hypoxic GBM-EVs induce generation of MDSC which secrete arginase to extracellular space leading to depletion of-arginine and inhibits activation and proliferation of T cells. T cells which appear to be right next to these arginase-secreting MDSC have dramatically reduced expression of granzyme B and marker of proliferation Ki67 (Grzywa et al., 2020). Hypoxic GBM-EVs were more potent in MDSC induction in comparison to normal non-hypoxic EVs *in vivo*. The transfer of miR-10a and miR-21 by EVs which were generated under hypoxia was confirmed. Transferred miRs activate generation and increase suppressive potential of MDSC and, hence, reduction of target proteins involved in RORα/IκBα/NF-κB and Pten/PI3K/AKT signaling cascades (Guo et al., 2018).

Taken together, the major populations involved in inhibition of T cell responses are macrophages and MDSC. This fact is directly confirmed by results of the study showing that removal of monocytic cells which are CD14^+^ from PBMC restores proliferation and activation of T cells, for instance, according to increase in CD69 and CD25 levels (Domenis et al., 2017).

### Dendritic cells (DC)

DC play a key role in induction of anti-tumor response because they are main professional antigen-presenting cells. In cerebrospinal fluid of GBM patients, the number of DC is higher than in healthy donors. These DC take up twice less EVs than monocytes but 3 times more than DC from peripheral blood (Harshyne et al., 2014). The effect of GBM-EVs on DC is not uniform. Published reports show that, despite increased numbers and activated status of DC from cerebrospinal fluid of GBM patients, most of these cells cannot effectively present tumor antigens. In some cases, GBM-EVs strongly decreased antigen-presenting potential of DC (Wang et al., 2020). At least one study demonstrated the inhibition in antigen processing and presentation in peripheral DC mediated by LGALS9^+^ from GBM-EVs isolated from primary GBM (Wang et al., 2021b). GBM-EVs bind Siglec-9, inhibitor of immune response, presented on DC in significant amounts. Surprisingly, another DC-specific molecule CD209 or DC-SIGN (Dendritic Cell-Specific Intercellular adhesion molecule-3-Grabbing Non-integrin, DC-SIGN, CD209) which mediates endocytosis and picking of antigens and activation of CD4^+^ and CD8^+^ T cells was not involved (Dusoswa et al., 2019).

The study by Sheybani and colleagues demonstrates the influence of DC by EVs from apoptotic GL261 GBM cells which were subjected to hypothermic conditions. Normal, non-apoptotic EVs from GBM suppressed DCs’ activation according to reduction of IL-12p70 production. However, treatment of these suppressed DC with hypothermic apoptotic EVs restored production of IL-12p70 to close to normal levels (Sheybani et al., 2020). This finding points to the possibility that, depending on conditions, GBM-EVs could inhibit or, oppositely, increase DC functions. This feature of DC could be useful in terms of anti-tumor immune responses activation, especially for specific anti-tumor T cells (Hao et al., 2007). One possible application of DC in clinical treatment of GBM will be discussed in the paragraph «Future perspectives of EV-based GBM treatment».

## Extracellular vesicles in diagnostic and prognosis of GBM

EVs are involved in most pathological processes in cancer. The distribution of GBM-EVs not only promotes the tumor immune escape, but also can change the tumor cells themselves due to their specific cargo. Thus, glioblastoma-derived vesicles have been shown to be involved in anti-tumor therapy resistance. Studies have reported that tumor EVs could transport non-coding RNAs and thus influence on the status of recipient cells. The presence of hypoxia-associated molecules in patient-derived vesicles such as AHIF and miR-301a was correlated with radiotherapy resistance of glioblastoma cells (Dai et al., 2019). Overexpression of macrophage movement inhibitory factor in glioma EVs from patients is related with TMZ resistance and can enhance therapy resistance in TMZ-sensitive glioma cells (Wei et al., 2021). The levels of vesicular miR-151a, miR-21 and miR-221 are negatively correlated with chemotherapeutic response of patients (Zeng et al., 2018; Mooij et al., 2020; Cheryl et al., 2013). The expression of SBF2-AS1 was significantly increased both in TMZ-resistant cells and in vesicles *in vitro* and *in vivo* (Zhang et al., 2019). Different circRNAs such as circNFIX and circ-METRN were reported to enhance radio resistance and progression of glioblastoma (Wang et al., 2021a; Ding et al., 2019). Vesicular miR-148a levels were higher in GBM patients compared with healthy donors so miR-148a can act as a GBM marker (Cai et al., 2018). Tumor-inhibiting miR-375 was downregulated in GBM patients. Lower miR-375 levels were also typical for vesicles from peripheral blood of GBM patients and correlated with poor prognosis (Xu et al., 2021b). MiR-944 could define tumor malignancy as high-grade gliomas express lower levels of miR-944 and correlate with lower overall survival of patients (Jiang et al., 2021; Guo et al., 2022). PD-L1^+^ vesicles were also found in peripheral blood and PD-L1 level was positively correlated with tumor size (Shenoy et al., 2021; Vautrot et al., 2021). These features of the EVs composition and the correlation between the expression levels and clinically significant indexes such as patient therapy response prediction and prognosis could not be unnoticed. EVs were suggested to use as biomarkers and there are several reasons for this.

Diagnosis of GBM is dependent on result of neuroimaging and tissue biopsies. However, neuroimaging such as magnetic resonance imaging (MRI) detects only well-developed brain tumors and contrast can change during chemotherapy leading to incorrect interpretation of image. Tumor biopsies can cause brain swelling and hemorrhage (Azam et al., 2019). One more problem is changing in MRI image indicating the tumor progress, but not necessarily accompanying by it. So-called pseudoprogression is a temporally local tumor necrosis resulting in tissue inflammation and occuring in response to radiotherapy. The incorrect interpretation of MRI image is problematic in terms of further treatment decisions for the patient (Strauss et al., 2021; Tankov and Walker., 2021). As a result, new diagnostic methods should be developed to solve these problems. Extracellular vesicles from GBM could be a promising biomarker, suitable not only for GBM presence confirmation, but also patient prognosis. Compared to most tumors, other circulating biomarkers such as circulating tumor cells or cell-free nucleic acids are of little use in case of brain tumors because of blood-brain barrier (Shi et al., 2020). However, EVs can cross the blood-brain barrier presumably using transcytosis (Banks et al., 2020; Ridder et al., 2014), so EVs could be found in biological fluids. Non-invasive liquid biopsy of blood or cerebrospinal fluid (CSF) could be used for GBM EV analysis (Alix-Panabières and Pantel., 2021).

There are a variety of methods to isolate vesicles from biological fluids. Ultracentrifugation procedure allows isolating vesicles with good yield and acceptable purity for subsequent functional studies (Ludwig et al., 2019). However, this method requires an expensive special equipment (ultracentrifuge) and a lot of time. Moreover, the vesicles obtained tend to aggregate or damage. The recommended volume of the beginning sample is not less than 25 mL, which is unreachable for biological fluids (Takov et al., 2018). These facts make this method more suitable for laboratory use than for clinical applications.

Ultrafiltration is also promising technique which allow to isolate the particles of determined size depending on the diameter of the pores in the selected membrane (Zhang et al., 2021). It seems to be less complex and not so time-consuming than ultracentrifugation. Dependence on the type of filter is the main difficulty of ultrafiltration. Furthermore, the final samples contain much more impurity non-vesicular proteins compared with samples isolated by centrifugation. Nevertheless, the combination of these two methods can solve the problem of aggregation and contamination (Lin et al., 2019), but it is still not applicable in clinics.

The commercially available isolation kits simplify the isolation of vesicles. These kits were shown to produce pure EVs with high yield for a short time from a sample less than 1 mL in volume. No expensive special equipment is needed, but isolation kits based on polymers change vesicles surface. It causes vesicle aggregation and impossibility to separate GBM EVs from the total mass (Sjoqvist et al., 2020).

Another isolation method is immunoaffinity capture. Antibody-coated magnetic beads or nanoparticles are used to separate vesicles with determined surface markers (Yousif et al., 2021). A new Aethlon ADAPT™ system (adaptive dialysis-like affinity platform technology) is a promising method for vesicles capture and removing (Qian et al., 2022). It consists of affinity agents or antibodies immobilized in the separator cartridges (Basu and Ghosh., 2019). However, definition and selection of the most specific GBM-markers is needed for future clinical applications of this system. It is also important to easily remove vesicles from antibody-coated matrix unless the vesicles would be damaged and unsuitable for further studies.

Size-exclusion chromatography is the most perspective vesicle isolation method, which is based on differential elution of biological fluid components depending on their size, shape, and molecular weight from stationary porous phase. It provides to obtain a very pure EV sample with a high yield from a low volume of fluid for a short time (Sidhom et al., 2020). However, the unwanted interactions between molecules in samples and stationary phase should be avoided. If the particles are not perfectly spherical, it may elute at a different stage than other spherical vesicles. There difficulties could lead to impurities in samples. Nevertheless, the application of this method is becoming more common (Guan et al., 2020).

Circulating EVs incapsulate specific GBM proteins, miRNAs, mRNAs, DNA and preserve them from proteases, so studying of vesicle-carried molecules could give more accurate results (Pathania et al., 2021). The analysis of vesicular profiles can give information about type, origin, differentiation and malignancy of brain tumor and also predict the therapeutic response and patient prognosis. RNAs described above are promising molecules to reach these goals (Shi et al., 2020). The main difficulty is to distinguish GBM EVs from other EVs in biological fluid. Surface EV proteins such as CD44 and CD133 could serve as biomarkers for GBM EVs because GSC express these tetraspanins on cell surface. GSC are also responsible for tumor metastasis and therapy resistance, which makes CD44 and CD133 important protein markers. Several GBM-associated vesicular components such as EGFRvIII, mutated isocitrate dehydrogenase 1, miR-151a and miR-21 are also interesting candidates for markers (Ludwig et al., 2022).

The analysis of EVs consists of quantitative and qualitative characterization. The most frequently used in laboratory methods (cryo-electron microscopy, nanoparticle tracking analysis, electron microscopy) are not suitable for clinics because of complexity of procedure and expensive special equipment needed (Nawaz et al., 2014). For quantitative analysis dynamic light scattering could be used due to wide size detection range and relative simplicity of method. Size analyzers are more available equipment in comparison with particle imagers used for nanoparticle tracking analysis (Tiwari et al., 2020). The commercially available protein assay kits are also could be used to determine the approximate amount of EVs in a sample by the concentration of protein (Nguyen et al., 2020). However, this method is inaccurate because of impurity proteins occurs, but seems to be attractive due to its cheapness and simplicity. The calibration for specific analysis conditions or kits measuring the determined proteins could bring this method closer to clinical application. Available qualitative methods include western blot, ELISA and flow cytometry (Nawaz et al., 2014). Molecular methods such as qRT-PCR or ddPCR are suitable for detection in EVs from blood or CSF and showed a high specificity and sensitivity (Azam et al., 2019).

The clinical application of EVs would simplify diagnosis and choice of therapy. However, some difficulties need to be solved first. For the beginning, the obtaining of EV sample with high purity is a very challenging task - there is no satisfactory isolation method that could provide good yield and high purity of samples and not requiring expensive equipment or time-consuming procedure. It may lead to low efficiency of the assay or false-positive results because of technical limitations in removing of contaminating proteins (Sidhom et al., 2020). Size-exclusion chromatography and immunoaffinity capture are the most promising methods for clinical application. As described above, the usage of immunoaffinity capture is limited by absence of the data about specific GBM-markers and impossibility to remove antibodies (Guo et al., 2022). So, specific GBM EVs surface markers are still needed to be defined and selected carefully. Next, not all markers could be easily identified in both EVs from blood and CSF because of differences in characteristics of EVs from different sources and patients. Sensitivity and specificity of the assays in early diagnostics and prognosis should be improved. The problem of impurities in size-exclusion chromatography could be solved by coupling of methods (Guan et al., 2020), so a lot of work has to be done to prepare a mixed protocol and to select optimal conditions.

## Future perspectives of EV-based GBM treatment

As described above, tumor EVs could be a reason of tumor progression and consequent irreparable damage to the entire body of the patient. They could also negatively influence the efficiency of therapy. It is obvious that elimination of GBM-EVs could improve the physical and mental state of patients and change the course of treatment. To minimize damage from vesicles, two strategies were suggested: blocking or inhibiting the release or uptake of tumor EVs.

To inhibit vesicles release, proton pumps inhibitor can be used (Federici et al., 2014). RAB27A and RAB27B proteins play an important role in vesicle biogenesis and can act as a target in cancer therapy. RAB27A inhibitors were shown to have a therapeutic potential in cancers (Zhang et al., 2020). Research on inhibition of glioma vesicles is at an early stage. MCT1, CD147, annexin A1 and VEGF-A were found to be a potential anti-glioma targets as silencing reduced vesicle release (Thakur et al., 2020; Treps et al., 2017; Vecchi et al., 2021). GW4869 is believed to be a possible anti-cancer therapeutic agent. GW4869 is a dihydroimidazolamide compound that is used as a specific inhibitor of neutral sphinogomyelinase which prevents the ceramide-dependent budding of MVBs and release of exosomes from MVBs (Peng et al., 2022). GW4869 was shown to reduce the levels of extracellular vesicles in serum and brain in a mouse model of Alzheimer disease (Broekman et al., 2018), so it probably should be tested in glioblastoma model.

Another way to influence the communication of tumor EVs and microenvironmental cells is uptake inhibition. The involvement of different glycoproteins on the vesicular membrane as well as the recipient cell is supposed in this process. The crucial role of heparanase and heparan sulfate proteoglycans in biogenesis of EVs has been confirmed (Nicholson et al., 2019). It was reported that the uptake of EVs from oral squamous cell carcinoma *in vivo* was blocked in presence of heparin (Sento et al., 2016). Christianson et al obtained the same result on model glioblastoma cell line U87 (Christianson et al., 2013). Heparin prevents interaction between vesicles and recipient cells on the surface ligand binding stage. It is supposed that heparin binds to cell receptors and interferes EV-recipient cell binding. Another mechanism of heparin action is that heparin causes aggregation of vesicles (Atai et al., 2013). Finally, heparin treatment helped to avoid monocyte reprogramming during incubation with patient-derived glioblastoma vesicles (Himes et al., 2022). Unfortunately, studies on heparin in glioblastoma model *in vivo* were not found in literature.

Another group of vesicle production inhibitors includes inhibitors of clathrin- and caveolin-induced endocytosis. Dynasore inhibits dynamin-2, which is essential for cell membrane curvature changing (Thakur et al., 2021b). Chlorpromazine binds surface receptors and inhibits formation of clathrin-coated pits. Blocking of phosphatidyl serine with annexin V also inhibits the EV uptake into microglia and other recipient cells (Pancholi et al., 2022).

ExoBlock is a novel phosphatidyl serine-binding molecule, which could be used to eliminate immunosuppressive vesicles from tumor microenvironment. It has been shown that ExoBlock treatment inhibited melanoma development *in vivo* and recovered T cell function including clonal expansion and IFN-γ production. Vesicular phosphatidyl serine was shown to reprogram macrophages to immunosuppressive phenotype. ExoBlock could change the polarization of macrophages to pro-inflammatory phenotype (Bhatta et al., 2021; Shenoy et al., 2021). Probably, this reagent or its counterparts will be useful in case of glioblastoma.

Immune checkpoint inhibitors could also be useful in cancer treatment. Anti-PD-1 and anti-PD-L1 antibodies have shown to be very promising in metastatic melanoma treatment (Chen et al., 2018). In case of glioblastoma immune checkpoint inhibitors did not demonstrate a survival benefit in phases 2 and 3 of clinical trials. It is believed that glioblastoma cells have a lower expression of PD-L1 and TILs have lower expression of PD-1 compared to melanoma (Ho and Ho, 2021; Lawler et al, 2020). One more reason is absence of tumor-specific T cells, which may be present in peripheral blood, but not in tumor microenvironment (Lee, 2021).

Tumor-derived extracellular vesicles also can be used in anti-cancer vaccines. The ability of EVs from tumors to inhibit anti-cancer immune responses makes it impossible to use them directly in vaccines. Moreover, EVs also have been reported to bear self-recognition molecules MHC-1 on their surface (Basu and Ghosh., 2019). Therefore, the usage of patient-deriver EVs in vaccines is considered to have no immunologic effect. Antigen-presenting cells could be a suitable intermediate between tumor EVs and activation of anti-tumor immunity. For instance, DCs were shown to produce specific dendritic cell-derived vesicles when incubated with tumor vesicles. This DC vesicles activated antitumor CD8^+^ T cells *in vivo* (Hao et al., 2007). The EV-based vaccines are currently being tested for melanoma and non-small-cell lung carcinoma cases, but the development of anti-glioma DS vesicles vaccine is also possible soon.

As described above, some studies showed the inhibitory effect of glioma EVs on DCs (Wang et al., 2021b). It could be a serious difficulty for application of EVs in anti-glioma vaccines. Dusoswa et al identified the presence of sialic acid and the absence of DC-SIGN ligands on the surface of glioblastoma EVs as the main reasons of inhibitory effect. The desialylation of EVs and the insertion of carbohydrate antigen palmitoyl-Lewis^Y^ led to more than four-fold increase in the EVs uptake by DC. The modification of tumor EVs for DC targeting could solve the problem of DC inhibition by glioma EVs and accelerate the development of EV-based anti-glioma vaccine (Dusoswa et al., 2019).

All these strategies are very perspective, but they have been applied only in preclinical studies. The main difficulty in release-uptake inhibitors usage in therapy is the non-selectivity of action. The inhibitors do not distinguish tumor-specific extracellular vesicle interactions from normal physiological processes (Bhatta et al., 2021). Much work will be needed to explore safe and effective routine clinical applications. The application of glioma EVs in vaccines could make some new difficulties too. The heterogeneity and the variety of brain tumors raises a question of EV sample selection. The vaccination with DC vesicles produced after incubation with one type of glioma EVs does not exclude the appearance of brain tumor characterized with another subtype, different surface and inner markers, etc. Nevertheless, considering numerous difficulties with glioma treatment and impossibility of full recovery, the approach avoiding the appearance of brain tumor, especially glioblastoma, could seem to be more attractive.

## Conclusion

In conclusion, it should be stressed that we are still far from understanding of intricate and extremely complex interplay between various factors forming immune suppressive TME. Heterogeneity of tumor cells, which is especially important in the case of GBM, interactions with tumor stroma, including immune cells of different origin and function, further complicate the task. Limitations of animal models of human GBM and lack of genetic and molecular instruments that would allow depletion of tumor EVs and/or definitive dissection of mechanisms utilized by EVs to transfer molecules/information slow down the progress in the field. On the positive side, tumor-derived EVs, especially, from GBM patients are recognized now as essential factor that may adversely influence the prognosis and efficacy of anti-tumor therapy. Other clinically useful feature of the GBM-EVs is their unique molecular signature which will have increasing value in non-invasive and early GBM diagnostics.

## Glossary

ADP: Adenosine diphosphate
Akt: Protein kinase B
ALG: Apoptosis-linked gene 2
Alix: ALG-2-interacting protein X
AMP: Adenosine monophosphate
AS1: Antisense RNA 1
ATP: Adenosine triphosphate
Bcl-xL: B cell lymphoma-extra large
CBTRUS: The Central Brain Tumor Registry of the United States
CCL12: Chemokine ligand 12
CCR4: C-C chemokine receptor type 4
CD: Cluster of differentiation
CLTC: Clatrin
CNS: Central nervous system
CSCs: Cancer stem cells
CSF: Cerebrospinal fluid
CTLA-4: Cytotoxic T-lymphocyte-associated protein 4
DC: Dendritic cell
DC-SIGN: Dendritic Cell-Specific Intercellular adhesion molecule-3-Grabbing Non-integrin
EGF: Epidermal growth factor
EGFR: EGF receptor
EGFRvIII: EGFR variant III (mutated)
EV: Extracellular vesicles
FasL: Fas ligand
FoxP3: Forkhead-box P3
GBM: Glioblastoma
GBM-EVs: EVs from GBM
GSC: Glioblastoma stem cell
HERV: Human endogenous retroviruses
HLA: Human leucocyte antigen
HLA-DR: Human leucocyte antigen DR isotype
HOTAIR: HOX transcript antisense RNA
HOX: Homeobox gene
HSP: Heat shock protein
IDO1: Indoleamine-2,3-dioxygenase 1
IFN-ɣ: Gamma interferon
IL: Interleukin
ITAM: Immunoreceptor tyrosine-based activation motif
IκBα: Nuclear factor of kappa light polypeptide gene enhancer in B-cells inhibitor, alpha
Ki67: Marker Of Proliferation
LDHB: Lactate dehydrogenase B
LGALS9: Galectin-9
lncRNA: Long non-coding RNA
LTRs: Long terminal repeats
MCP-1: Monocyte chemoattractant Protein 1
MDSC: Myeloid-derived suppressor cells
MHCII: Major histocompatibility complex class II
miR: MicroRNA
MRI: Magnetic resonance imaging
mRNA: Messenger RNA
MT1-MMP: Membrane type 1-matrix metalloproteinase
MVB: Multivesicular bodies
ncRNA: Non-coding RNA
NF-κB: Nuclear factor kappa B
PBMC: Peripheral blood mononuclear cells
PD-1: Programmed cell death 1
PD-L1: Programmed cell death ligand-1
PGK1: Phosphoglycerate kinase 1
PI3K: Phosphatidylinositol-4,5-bisphosphate 3-kinase
piRNA: Piwi-interacting RNA
PRKDC: DNA-dependent protein kinase, catalytic subunit
PTEN: Phosphatase and tensin homolog
ROR1: Regulator of reprogramming 1
RORα: Retinoic acid receptor-related orphan receptor alpha
SBF2: SET Binding factor
SHP: Src homology region 2 domain-containing phosphatase-1
Siglec-9: Sialic acid-binding Ig-like lectin 9
SINEs: Short interspersed nuclear element
snoRNA: Small nucleolar RNA
snRNA: Small nuclear RNA
STAT5: Signal transducer and activator of transcription 5
TCR: T cell receptor
TGF-β: Transforming growth factor beta
TILs: Tumor infiltrating lymphocytes
TIM-3: T cell immunoglobulin and mucin domain containing-3
TME: Tumor microenvironment
TMZ: Temozolomide
TNF: Tumor necrosis factor
TRAIL: TNF-related apoptosis-inducing ligand
Treg: Regulatory T cells
Tsg101: Tumor susceptibility gene 101
VEGF: Vascular endothelial growth factor
VIME: Vimentin
