# Supplementary material for: Effects of glioblastoma-derived extracellular vesicles on the functions of immune cells
Source: Front Cell Dev Biol. 2023 Mar 7;11:1060000. doi: 10.3389/fcell.2023.1060000 (PMC10028257; doi:10.3389/fcell.2023.1060000)
Supplement: Supplementary file 1 [file Table1.DOCX]

Table 1. List of RNAs isolated from glioblastoma EVs and their effects on target cells.

| RNA | Type | Role in glioma progression | Reference |
| --- | --- | --- | --- |
| miR-10a | miRNA | Implicated in temozolomide resistance | doi:10.1016/j.canlet.2010.04.013 |
| miR-10b | miRNA | Associated with tumor invasion | doi:10.1002/ijc.24522 |
| miR-21 | miRNA | Oncogene that targets components of p53 and TGF-β pathways in microglia | <https://doi.org/10.1111/j.1582-4934.2008.00556.x> |
| miR-23a | miRNA | Promotes glioblastoma invasion | https://doi.org/10.1038/s41392-018-0033-6 |
| miR-23b | miRNA | Glioma survival and invasion | doi:10.1093/neuonc/nos122 |
| miR-27a-3p | miRNA | Involved in M2 macrophage polarization | https://doi.org/10.1038/s41420-022-01035-z |
| miR-30 family | miRNA | Inhibit mitochondrial fission and the consequent apoptosis | <https://doi.org/10.1371/journal.pgen.1000795> |
| miR-100-5p | miRNA | Overproduction lowers tumor mass and stem cell markers | <https://doi.org/10.3390/biomedicines10010112> |
| miR-145 | miRNA | Hypoxia-induced inhibitor of glioma migration | <https://doi.org/10.1093/neuonc/not090> |
| miR-193a | miRNA | Hypoxia-induced inhibitor of glioma migration and invasion | https://doi.org/10.1186/1471-2164-15-686 |
| miR-210 | miRNA | Hypoxia-induced angiogenic activity | https://doi.org/10.1186/1471-2164-15-686 |
| miR-221 | miRNA | Downregulation of p27Kip1, a key negative regulator of the cell cycle | <https://doi.org/10.4161/cc.6.16.4526> |
| miR-222 | miRNA | Downregulation of p27Kip1, a key negative regulator of the cell cycle | <https://doi.org/10.4161/cc.6.16.4526> |
| miR-376c | miRNA | Impairs in TGF-β signaling | 10.1161/HYPERTENSIONAHA.111.203489 |
| miR-451 | miRNA | Downregulates the level of tumor suppressor c-Myc | <https://doi.org/10.1093/neuonc/nov244> |
| miR-1246 | miRNA | Promotes migration and invasion | https://doi.org/10.1038/s41374-020-00522-0 |
| miR-1275 | miRNA | Stemness maintenance | doi:10.1002/1878-0261.12525 |
| miR-4448 | miRNA | Maintains stemness of glioblastoma stem cells | <https://doi.org/10.1002/jcp.24614> |
| Let-7 family | miRNA | Promotes glioblastoma  migration and invasion | <https://doi.org/10.1186/s13046-018-0841-0> |
| HOTAIR | lncRNA | Angiogenic activity | PMC5714785 |
| LINK00470 | lncRNA | Regulator of proliferation of glioblastoma cells and autophagy | https://doi.org/10.1186/s12935-021-01825-y |
| ROR1-AS1 | lncRNA | Inhibitor of tumor suppressor miRNA miR-4686 | [10.2147/IJN.S271795](https://doi.org/10.2147%2FIJN.S271795) |
| SBF2-AS1 | lncRNA | Implicated in temozolomide resistance | https://doi.org/10.1186/s13046-019-1139-6 |
| Alu | RT | Mutation driver | https://doi.org/10.1038/ncomms1180 |
| HERV | RT | Mutation driver | https://doi.org/10.1038/ncomms1180 |
| L1 | RT | Mutation driver | https://doi.org/10.1038/ncomms1180 |
| LTR | RT | Gene silencing in recipient cells | <https://doi.org/10.4161/rna.25281> |
| SINE | RT | Gene silencing in recipient cells | <https://doi.org/10.4161/rna.25281> |

RT-retrotransposion, miRNA – microRNA, lncRNA -long non-coding RNAs
